# Use of Hyaluronic Acid–Based Biological Bilaminar Matrix in Wound Bed Preparation: A Case Series

**Published:** 2018-04-26

**Authors:** Richard Simman, Walid Mari, Sara Younes, Michael Wilson

**Affiliations:** ^a^Dermatology; ^b^Pharmacology and Toxicology, Wright State University Boonshoft School of Medicine, Dayton, Ohio

**Keywords:** wound, healing, Hyalomatrix, hyaluronic acid, extracellular matrix

## Abstract

**Objective:** Hyalomatrix, a matrix that consists of esterified hyaluronic acid, covered with a removable, semipermeable silicone top layer, was used to generate granulation tissue in a series of 12 serious, surgical wounds of different etiologies. **Methods:** Many defects showed exposed muscle, tendons, and/or bone. After thorough debridement, the matrix was applied, in a number of cases, combined with negative pressure wound therapy. **Results:** All wounds developed granulation tissue in and on top of the matrix. Nine wounds were then closed with a split skin autograft. There was no graft failure, and all wounds showed complete reepithelialization. Three wounds healed by secondary intention and also reached complete healing. **Conclusion:** This case series shows a strong trend for Hyalomatrix to play an important role in supporting wound healing in complex, surgical wounds.

As a surgical rule and represented in the well-documented recommendations of staged wound healing,^1^^,^^2^ removal of devitalized tissue is an essential part of wound treatment and the initiation of wound repair,^3^^-^5 whether the necrosis is caused by thermal or mechanical injury, infection, or any other reason. Unless the lesion is small enough to allow for relatively rapid reepithelialization by secondary intention, the lesion then should be covered by an autograft such as a split skin graft or a flap.

Deep wounds pose a problem with regard to graft take: although grafting directly on clean, healthy muscle is possible,^5^ direct coverage of tendon and bone is difficult without the development of granulation tissue over these structures.^6^ In addition, such practice may lead, upon healing, to a depression in the skin surface, which may be cosmetically undesirable.

Replacement of lost extracellular matrix (ECM) in deep wounds has been proven to be beneficial with regard to the development of granulation tissue and the quality and speed of healing.^7^^,^^8^ Many matrices are now available, and all aim at replacing the lost ECM with a matrix that will allow and encourage the production of a granulating wound bed.^9^^-^12

Some of these products are based on esterified hyaluronan (also known as hyaluronic acid [HA]), which is a regular and abundant compound of the ECM and plays several roles in homeostasis and healing.^13^^-^16 Hyalomatrix (Medline Industries, Inc., Northfield, Ill)* (eHAM) is a biodegradable dermal matrix contact layer made of Hyaff (Medline), an esterified form of HA. The dressing has a protective outer silicone layer. The matrix acts as a scaffold for the creation of a granulating wound bed, which can be grafted upon after removal of the top layer. eHAM has been used successfully as an ECM in different types of wounds.

This retrospective case series describes the use of eHAM in subjects with full-thickness lesions of different etiology, but primarily because of serious soft tissue infections, and presents 2 typical complex cases.

## METHODOLOGY

eHAM was used on a trial basis in our clinic from August 2015 through September 2016, and all patients treated with eHAM were enrolled in the case series. Patients with different kinds of indications were referred to our clinic. One patient suffered from a complicated free latissimus dorsi flap with skin graft after reconstruction of an excised and radiated liposarcoma of the left leg. For all other patients, wound infection with necrosis and dehiscence, either primary or after surgical intervention, was the main indication for referral (Table 1). All wounds underwent rigorous surgical debridement: this included the removal of osteomyelitic bone and necrotic soft tissue including tendons, if and when present and indicated. In 4 cases, multiple debridement sessions were necessary to remove all necrosis. When the wound was properly debrided, eHAM was applied. Once tissue growth (granulation tissue) into the matrix was obtained, the silicone top layer was removed (although in 2 patients, the top layer came off by itself) and healing progress was assessed. If there was an indication for a split skin autograft, as judged by the clinical appearance of the wound, a graft was taken and applied to the recipient site. Depending on the location and type of lesion, negative pressure wound therapy ([NPWT], VAC; KCI/Acelity, San Antonio, Tex) or suturing with 3/0 chromic catgut was used for graft fixation.

eHAM that was bolstered with NPWT was covered with Xeroform (DeRoyal, Powell, Tenn). Lesions for which NPWT was not used were covered with Aquacel Ag (ConvaTec, Bridgewater, NJ) or Xeroform, depending on the amount of exudate. Dressings were changed twice a week after the first postoperative dressing. All patients received 1 dose of antibiotics per our operation protocol unless antibiotics were already initiated by infectious disease colleagues.

### DEMOGRAPHICS

**Figure 1:**
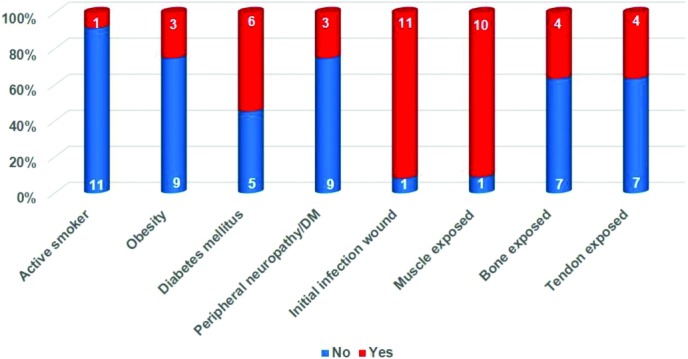
Compromising factors, wound characteristics. DM indicates diabetes mellitus.

Twelve patients, 8 male and 4 female, participated in this study. The average age was 52.4 years (min: 26, max: 76). The diagnoses and locations of the lesions are listed in Table 1. One patient (9%) was an active smoker, 3 patients (25%) were obese, and 7 patients (58%) suffered from diabetes mellitus type I or II.^†^ Three of these (25% of total) also suffered from peripheral neuropathy (Fig 1).

### Case 1

A 68-year old woman with a history of diabetes and status post disarticulation of the left hip due to necrotizing fasciitis was referred to the long-term acute care (LTAC) hospital for wound care, nutritional support, and intravenous antimicrobial therapy. The large wound was treated with multiple bedside debridements and NPWT. The wound stalled and several bony areas became exposed, most notably the acetabular region, anterior superior iliac crest, and the os pubis. No granulation occurred over these areas.

**Figure 2:**
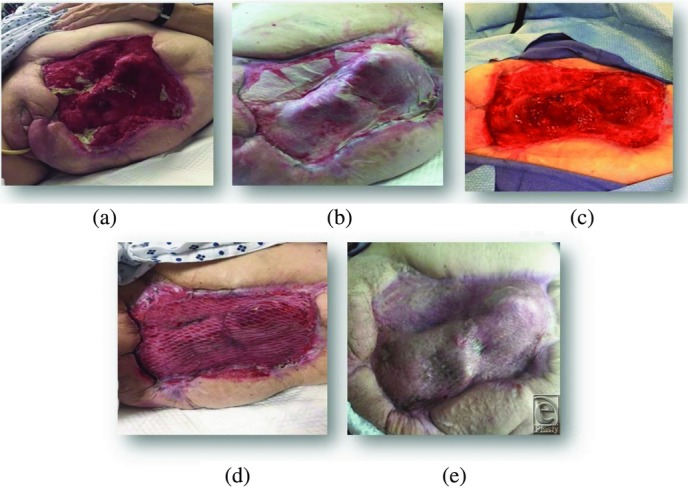
Status post disarticulation of the left hip in a 68-year-old female with a history of diabetes. Disarticulation was necessary because of extensive necrotizing fasciitis. The wound shows exposed bone.

The patient was taken to the OR and underwent debridement (Fig 2*a*) of her wound, including bone and ligaments. eHAM was applied (Figure 2*b*) in combination with NPWT. After a period of 3 weeks, the top silicone layer of the matrix and the eHAM remnants were removed (Fig 2*c*). A meshed split skin autograft was applied (Fig 2*d*) and was secured with NPWT for 2 weeks. The skin graft showed 100% take, and after 2 weeks the wound was completely healed (Fig 2*e*).

### Case 2

**Figure 3:**
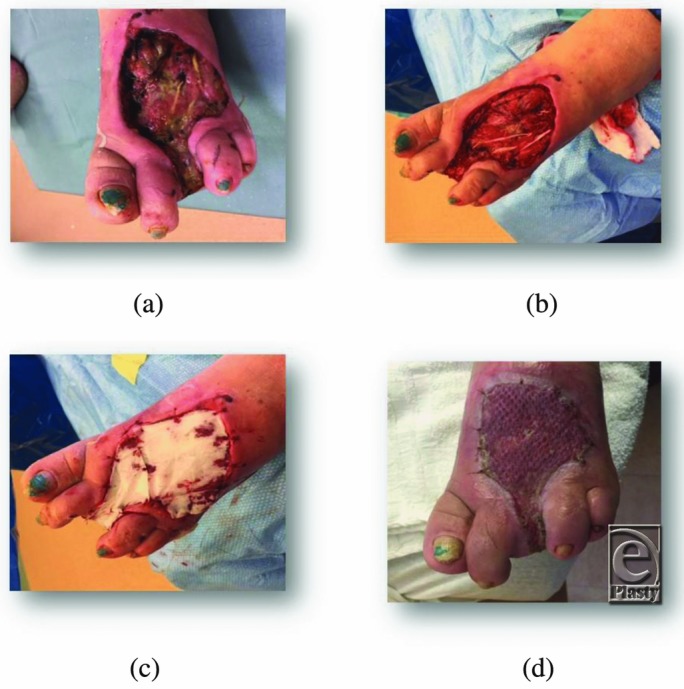
A 58-year-old woman with a necrotizing soft-tissue infection of her left foot and advanced osteomyelitis of her left middle toe.

A 58-year old woman with diabetes and peripheral neuropathy was admitted through the emergency department to the hospital with a necrotizing soft tissue infection of her left foot and advanced osteomyelitis of her left middle toe. After receiving surgical debridement, including a toe amputation, and intravenous antibiotic therapy in combination with proper wound care, she was admitted to the LTAC hospital with residual soft tissue necrosis including the extensor tendons (Fig 3*a*).

The patient was taken to the OR for surgical debridement of her foot including tendons (Fig 3*b*). eHAM was applied (Fig 3*c*) over the defect to cover her tendons and combined with NPWT. After 1 week, the silicone layer came off and the wound was dressed twice daily with wet and moist dressing using quarter-strength Dakin's solution. The affected extremity was kept elevated until it was ready for skin grafting 2 weeks later. A meshed split skin autograft was applied to the wound with NPWT for a week to secure the graft. During the following week, dressing changes were done daily until the wound was completely healed (Fig 3*d* shows the wound 2 weeks postgrafting).

## RESULTS

**Figure 4:**
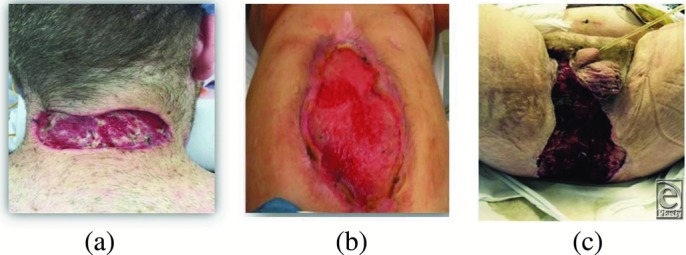
Clinical examples of different types of lesions. (a) Abscess due to ingrowth hair. Postincision/excision in a healthy 26-year-old man. (b) Post-ventral hernia repair with post-operative infection and dehiscence in a 67-year-old man. (c) Fournier's gangrene in a 63-year old man with diabetes mellitus and obesity. Postoperative view.

Lesions ranged from relatively small in healthy patients (Fig 4*a*) to relatively large but “simple” (Fig 4*b*) to large and complex (Fig 4*c*). Muscle was exposed in 11 patients (92%) and tendon and/or bone in 5 patients (42%). In 11 patients (92%), wound infection was the primary reason for debridement and application of eHAM (Fig 1).

In all patients, extensive sharp debridement and excision were performed in the operation room (OR). For most wounds, debridement could be limited to 1 session but multiple sessions were necessary for some patients (n = 4; 25%), where a good wound bed could not be reached within 1 session. In 10 patients (83%), 1 application of eHAM suffices. One patient (8%) required 2 applications and 1 patient (8%) needed 4 applications.

NPWT was used in 9 patients (75%) for securing the eHAM, and sutures were used in the remaining 3 cases (25%). On average, the period a wound was (pre) treated with eHAM was 22.6 days (min: 14, max: 56).

In 9 cases (75%), the wound was grafted with a split skin graft. In 8 of these cases (89%), the graft was fixated with NPWT and in 1 case (11%) with a splint. In 3 cases (25%), healing per secondary intention occurred.

All lesions reached complete healing. For all lesions that healed by secondary intention, time to reepithelialization was 42 days (n = 3). For those that were grafted, the average total healing time, measured from the initial application of eHAM, was 37.9 days (min: 21, max: 70).

## DISCUSSION

Tissue loss, whether through trauma, infection, or surgical causes, can be a major impediment to rapid wound closure. Infection per se as a complication in patients with impaired immunocompetence through local or systemic causes such as diabetes mellitus may lead to major tissue loss: infection in diabetic patients is one of the major reasons for nontraumatic amputations.^17^ Primary infections such as necrotizing fasciitis and Fournier's gangrene require radical excision of all infected and dead tissue, which, per definition, also leads to major tissue loss.^18^^,^^19^

With proper wound management, even large tissue defects may heal by secondary intention, but the process requires a large amount of time and, in fact, may stall. The use of split skin autograft is an easy way to obtain more rapid coverage, but such a graft requires a clean wound bed.

Several matrices are designed both to fill in gaps caused by tissue loss and to provide such a wound bed. Replacement of lost ECM has been proven to be beneficial with regard to the quality and speed of healing as proven in many indications.^7^^,^^8^ Burk et al^20^ were the first to develop and use an artificial ECM made out of collagen and glycosaminoglycan. Several other products now are available. Some contain cells, whereas others are acellular but provide a “dermal environment” that encourages repopulation with cells. All these products are designed to replace lost ECM and to produce a proper granulating wound bed^9^^-^12 and a proper wound bed.

Among these, matrices based on hyaluronan have been shown to be particularly effective. HA, an anionic, nonsulfated glycosaminoglycan, is a major component of the normal ECM.

It plays an essential role in all stages of normal postnatal wound healing. It provides wounds with a moist environment, stimulates angiogenesis,^14^ facilitates and directs cell proliferation,^13^ including fibroblast migration,^15^ regulates tissue hydrodynamics,^16^ stabilizes the newly built ECM,^21^ and regulates many other different processes involved in healing.^13^

HA also plays a major role in fetal wound healing, which is characterized by minimal inflammation and scarless repair.^22^^,^^23^ Comparison between postnatal and fetal wound healing has revealed differences in inflammatory response, cellular mediators, cytokines, growth factors, and ECM modulators^24^: among other properties, fetal wounds are characterized by high levels of HA and its stimulator(s) with more rapid, highly organized collagen deposition.^25^

HA is being used extensively in tissue repair such as for chondral and osteochondral lesions due to injury or other pathology^26^^,^^27^ and as a wound filler. Wound fillers vary extensively with regard to their level of cross-linking, particle concentration, immunogenicity, biofilm formation, and gel hardness, and (cross-linked) HA seems to provide a superior combination of durability within the skin and low immunogenicity.^28^ Consequently, HA is used widely in the correction of skin lesions, for indications such as acne repair, in the treatment of atrophic scars, and as a filler for depressed scars or contour deficiencies.^23^^,^^29^^-^31

In its native form, HA is difficult to handle and it has a short half-life, particularly in the presence of large amounts of hyaluronidases, as may be the case in stalled wounds.^32^ Esterification changes the physical properties of the HA, for example, rendering it longer lasting in a tissue environment without scission of the polymer chain and dissolution. The level of esterification can be adjusted using a proprietary technology^33^ where the end product is called Hyaff and is presented as fibers produced through the process of electrospinning. In a series of preclinical tests, Hyaff was shown to be appropriate as a scaffold for tissue engineering. The material's properties (vs native hyaluronan), in combination with improved residence time, were found to provide an expansion of the possible applications of HA in the biomedical field.^15^^,^^33^^-^35

eHAM is a biodegradable dermal matrix contact layer, made of Hyaff, which, postelectrospinning, is made into a soft, nonwoven mat of fine fibers. The dressing has an outer silicone layer that is semipermeable. This silicone layer provides physical, protective coverage of the underlying wound tissues and controls water vapor loss. The matrix acts as a scaffold for cellular colonization (eg, fibroblasts) and capillary ingrowth, creating a granulating wound bed. Once tissue has grown into the dressing and granulation tissue has formed, the silicone top layer can be removed by peeling off and the resulting wound bed can be grafted. The dressing has been used successfully as an ECM in several indications,^36^^-^39 thus contributing to the healing of very different types of lesions, including lesions with exposed bone and/or and tendons^40^^-^43 and infected diabetic foot ulcers.^40^ eHAM also has been used for the reconstruction of syndactyly^44^ and the revision of scars.^45^

The patients in this case series all had major tissue loss, many of them had exposed bone and/or tendons and/or muscle, and some of them were suffering from osteomyelitis. In addition, a number of patients suffered from diabetes mellitus and obesity or were active smokers, all conditions known to impede wound healing.^46^^-^49 In all cases, extensive debridement was followed by the application of eHAM to fill in tissue gaps and to provide a granulating wound bed as part of wound bed preparation. In 3 cases, the wounds continued to heal and reepithelialize on their own whereas the remainder of the wounds were autografted. All lesions achieved complete reepithelialization, on average, within 42 days for those that healed by secondary intention and, on average, in 37.9 days for those that were grafted.

## LIMITATIONS

A case series, as opposed to a comparative clinical trial, can only show trends and cannot be used to demonstrate superiority of a given product, other than via comparison with literature results on similar indications. Moreover, most case series are generated using a limited series of inclusion and exclusion criteria, making for a nonhomogeneous study cohort.

However, when results are very consistent, even when the indications and etiologies within the patient cohort are diverse, as is the case in the 12 patients demonstrated here, strong trends may be observed.

## CONCLUSION

In a series of 12 patients with a variety of surgical and, mostly, very serious and complex wounds, the combination of thorough debridement and the creation of a granulating wound bed through the application of eHAM allowed for rapid and complete reepithelialization. Of the 12 patients, 3 were treated exclusively with eHAM matrix until reepithelialization was complete. For the remaining 8 patients, eHAM proved efficacious in preparing the wound bed by enhancing granulation tissue formation and readiness for a split-thickness skin graft. There was no incident of graft failure observed in any of the cases, and all presented wounds, whether grafted or not, healed in 5 to 8 weeks from presentation.

The study clearly demonstrated a strong trend showing that eHAM provides a good wound bed for both healing by secondary intention and for split skin autografting without graft failure and contributed to rapid and complete healing of all wounds.

## Figures and Tables

**Table 1:** Diagnoses*

Patient no.	Etiology/primary surgical intervention	Diagnosis/indication for eHAM	Location
1 (case 1)	Nec. Fasc. leading to disarticulation in the hip	Extensive debridement acetabular region, anterior superior iliac crest, os pubis, ligaments	Left hip
2 (case 2)	Amputation toe	Extensive soft tissue necrosis dorsum	Left foot
3	Abscess	Postexcisional debridement due to necrotizing infection	Neck
4	TMA	Gangrenous forefoot after TMA. Osteomyelitis, failed HBOT	Right foot
5	CABG	Sternal wound dehiscence, soft tissue necrosis of the left breast	Sternum
			Left breast
6	Extended period of unconsciousness while kneeling	Full-thickness necrosis	Right knee
7	Fournier gangrene	Debridement + colostomy	Perineum
			Scrotum
8	Repair ventral hernia	Dehiscence, postoperative infection, debridement nonhealing for 1 y	Midline abdomen
9	CABG	Intravenous site infiltrate, soft tissue necrosis. Debridement	Right hand
10	Nec. Fasc. after dog bite	Amputation of all digits at the metacarpal joint	Left hand
11	Excision liposarcoma	Reconstruction with flap and radiation therapy. Subsequent flap dehiscence and 30 × HBOT sessions (failed to heal)	Left ankle
12	TMA	Stalled wound with osteomyelitis and failed HBOT	Right foot

*Nec. Fasc. indicates necrotizing fasciitis; TMA, transmetatarsal amputation; HBOT, hyperbaric oxygen therapy; and CABG, coronary artery bypass grafting.

## Footnotes

*Hyalomatrix. In the European Union, the matrix is called Hyalomatrix PA.

^†^Note that percentages may not add up to 100% due to rounding.
